# Workplace-based assessment in South African postgraduate medical training: A baseline survey

**DOI:** 10.4102/jcmsa.v2i1.88

**Published:** 2024-11-21

**Authors:** Tasleem Ras, Emma Daitz, Louis S. Jenkins, Jacques Janse van Rendsburg, Madeleine Muller, Veena Singaram, Richard Cooke, Sumaiya Adam, Dini Mawela, Gerda Botha, Thakadu Mamashela, Tashneem Harris, Eric Buch, Lionel Green-Thompson, Vanessa Burch

**Affiliations:** 1 Department of Family, Community and Emergency Care, Faculty of Health Sciences, University of Cape Town, Cape Town, South Africa; 2 Department of Family Medicine, Faculty of Health Sciences, Stellenbosch University, Stellenbosch, South Africa; 3 Department of Diagnostic Radiology, Faculty of Health Sciences, University of the Free State, Bloemfontein, South Africa; 4 Department of Family Medicine, Faculty of Health Sciences, Walter Sisulu University, East London, South Africa; 5 School of Clinical Medicine, University of KwaZulu-Natal, eThekwini, South Africa; 6 Department of Family Medicine and Primary Care, Faculty of Health Sciences, University of the Witwatersrand, Johannesburg, South Africa; 7 Department of Obstetrics and Gynaecology, Faculty of Health Sciences, University of Pretoria, Tshwane, South Africa; 8 Department of Paediatrics and Child Health, Faculty of Health Sciences, Sefako Makgatho Health Sciences University, Tshwane, South Africa; 9 Practice of Medicine, Faculty of Health Sciences, Sefako Makgatho Health Sciences University, Tshwane, South Africa; 10 Department of Forensic Pathology, Faculty of Health Sciences, University of Limpopo, Polokwane, South Africa; 11 Department of Otolaryngology, Faculty of Health Sciences, University of Cape Town, Cape Town, South Africa; 12 Colleges of Medicine of South Africa, Rondebosch, South Africa; 13 SA Committee of Medical Deans and Deanery, South Africa; 14 Faculty of Health Sciences, University of Cape Town, Cape Town, South Africa

**Keywords:** workplace-based assessment, competency-based medical education, postgraduate, LMIC, Africa

## Abstract

**Background:**

In line with international best practices, the South African Committee of Medical Deans, supported by the Colleges of Medicine of South Africa, has called for institutions educating medical specialists to start integrating workplace-based assessment (WBA) in 2025. Workplace-based assessment requires that clinical supervisors observe trainees in the real world of clinical practice, provide feedback and foster reflective practice, while foregrounding patient safety. Despite a large literature on WBA in the global north and an emerging literature in South Africa, a framework for WBA implementation, grounded in South African realities, does not exist. The study aimed to determine current WBA-related knowledge and practices among registrar educators.

**Methods:**

A cross-sectional observational study, using an online 5-point Likert scale questionnaire, explored current knowledge and practices of WBA-related activities. The questionnaire variables were categorical, and the data were analysed descriptively. Frequencies, proportions and appropriate graphics were used to present the data.

**Results:**

The key findings relate to relatively high levels of knowledge of what constitutes WBA (82.5% agreed that they had knowledge of WBA), juxtaposed against large variability in the levels of implementation of WBA practices.

**Conclusion:**

The study surveyed postgraduate supervisors in a low- and middle-income country (LMIC) context regarding their understanding and current practices of WBA. Self-reported knowledge levels were high while practices were variable

**Contribution:**

The study provides insights into areas to focus on, with the future development and implementation of a comprehensive WBA strategy in South Africa.

## Introduction

In line with international best practices, the South African Committee of Medical Deans (SACOMD), supported by the Colleges of Medicine of South Africa (CMSA), has called on institutions educating medical specialists to start integrating workplace-based assessment (WBA) from 2025. Workplace-based assessment requires that supervisors observe trainees in the real world of clinical practice^1^ and provide feedback, thereby fostering reflective practice.^2^ Emerging from and aligned with pre-existing competency-based medical education (CBME) practices, WBA seeks to build a connection between the measurement of competency and its implementation in the real world of clinical medicine.^3^ It encompasses a wide range of assessment strategies that collect and collate data on trainee competence. This information is used to provide developmental feedback in formative assessments and to make judgements in summative assessments. A comprehensive WBA framework encompasses training of faculty in the skills of workplace observation and assessment, standardised workplace formative assessments and regular feedback to trainees,^4^ streamlined processes of data collection and collation, and the formation of clinical competence committees to make summative decisions. An emerging literature supports the implementation of WBA in the South African context,^5,6^ although, to date, a framework or guideline for WBA implementation grounded in South African realities, does not exist.

A comprehensive framework for WBA incorporates four key dimensions^7^: the existence of clear outcomes, called Entrustable Professional Activities (EPA) in the WBA literature^8^; a broadly accepted format for workplace observation, feedback and assessment; the collection of data generated by workplace activities into a portfolio of learning; and the use of this collated data to make a summative decision about a trainee’s progress.^9^ Entrustable Professional Activities, which describe units of professional work trainees are expected to learn to do independently, have become one of the preferred frameworks for structuring the collation of WBA data. Entrustable Professional Activities integrate competencies, and best-practice guidelines are now available for writing EPAs.^8^ Rather than assigning marks in the early and formative stages of training and observation, EPAs use entrustment supervision scales which ask observers to reflect on two issues, namely ‘*What are we trusting trainees to do?*’ and ‘*How do we make decisions to trust or not trust?*’ Entrustment supervision scales use descriptors of the level of supervision trainees require to perform a task^10,11^ and provide valuable opportunities for formative feedback. As such their value cannot be understood within a simple binary pass or fail paradigm. This requires high levels of ‘assessment literacy’ among faculty, making faculty development a key consideration when implementing and sustaining WBA practices.^12^

Workplace observations, which generate data documenting the trainees’ learning journeys, can take many forms and can focus on any aspect of professional life covered within an EPA.^7^ To ensure that trainees are guided towards achieving independence within a particular EPA, there needs to be common understanding and purpose among supervisors when doing these workplace observations, again highlighting the importance of faculty development.^12^ This would ensure that the data captured as a product of the workplace learning encounter contributes to the trainees’ ongoing developmental trajectory.

The need to collect adequate data to create a useful portfolio of learning that maximises the trustworthiness (validity and reliability) of WBA decisions,^13^ has created an additional administrative burden,^14^ which can be partly addressed by using mobile devices and digital applications.^15,16^ These digital applications act as data repositories and in some instances, can analyse the data, link to the EPA framework and produce digital dashboards that provide live tracking of trainees’ performance.

Clinical Competency Committees (CCCs) are necessary for the summative decision-making phase of WBA.^9^ They comprise groups of experts, who review data generated by WBA activities and serve as high-stakes decision-makers (summative assessment) and interface with key stakeholders in the assessment process. As such they are also useful resources of knowledge, experience and expertise for training programmes. They enhance the assessment system by identifying gaps and redundancies and promoting faculty improvement.^9^ The primary purpose of CCCs, beyond making summative judgements about trainee progress, is to ensure patient safety and promote high-quality clinical care by identifying issues which can have a negative impact.^9^

The components thus described, namely, workplace observations within an EPA framework; a digital portfolio of evidence of learning; and a CCC that uses the portfolio evidence, constitute the broad outline of a WBA programme. There is no published literature in South Africa or continentally, and a paucity from within low- and middle-income countries (LMICs), that deals with this comprehensive programme. As South Africa embarks on a WBA implementation project, having a baseline understanding adds to what is known about WBA in the local context and provides evidence for strategic planning that will inform future work in this regard. This study aims to describe current WBA-related knowledge and practices in specialist training programmes in South Africa prior to a large-scale joint initiative driving the implementation of WBA by a loose consortium of key stakeholders.

## Research methods and design

### Study design

A descriptive cross-sectional study, using a mixed methods design, was conducted. The qualitative component has been previously published,^6^ while this article addresses the quantitative component only.

### Population and sampling

Postgraduate specialist training programme convenors at all participating institutions in South Africa formed the study population. All were purposively invited to participate. The estimated total population was 270, and we aimed to ensure that at least 80% (*n* = 216) would participate.

#### Inclusion criteria

- Academic appointee (part or full-time), with appropriate specialist qualifications in the relevant clinical discipline.
- Formally appointed at an academic institution as a postgraduate specialist training programme manager (part or full-time) *or* spends a significant amount of time (as deemed by the participant) managing a postgraduate specialist training programme *or* is involved at a faculty level in postgraduate specialist training programme governance.
- Agree to participate via the informed consent process.
- We did not apply any exclusion criteria.

### Recruitment

The project team included representatives from all institutions currently offering postgraduate specialist training in South Africa. Each institutional representative recruited participants from their respective institutions, using line management and governance structures. The project already had support from all 10 health sciences faculty deans in the country. Recruitment was conducted via an email link to an online survey using cloud-based services (Google Forms). This online form included an informed consent component that allowed invitees to indicate whether they were opting in or out. No incentives were offered for participation. The project lead monitored responses and communicated with institutional leads when response rates were low. Institutional leads reached out directly to potential participants, either in person, by phone or via email. Despite these efforts, some institutions still experienced low response rates. Most participants took between 10 min and 15 min to complete the questionnaire.

Data collection continued for a period of 5 months (October 2022 – February 2023). Because of time demands, the data collection phase was terminated before the target sample size was reached.

### Data collection

A novel online questionnaire (Online Appendix 1) explored current knowledge and practices of WBA-related activities. The questionnaire, comprising multiple items and a 5-point Likert-type scale, was initially compiled in English by the senior author, based on an in-depth review of the literature. This incorporated all key aspects of a WBA framework, with each item validated against the literature. Content validity was pursued by assembling a national panel of educational and clinical specialists, consisting of 11 members from diverse clinical disciplines and representing all local health sciences faculties. They reviewed the draft independently, drawing on their knowledge of the literature and their own contexts. Some items were removed because of duplication, and items dealing with the CCC were added. A total of 25 items were included in the final tool, with each item comprising a statement about an aspect of WBA with an accompanying 5-point Likert scale ranging from ‘Strongly Agree’ to ‘Strongly disagree’.

A pilot study was undertaken in July 2022 among nine programme directors not affiliated with this project. This pilot tested the online delivery of the questionnaire using Google Forms. After agreeing to participate, participants were emailed the link to the online questionnaire, which was prefaced by the informed consent form. After signing the form electronically, they were asked to complete the form and comment on the mode of delivery, clarity of questions being asked and its face validity. Additionally, they were asked to provide open-text suggestions for improvements. Several comments were received, which led to minor semantic changes but none to any of the items.

### Data analysis

The questionnaire variables were categorical, and the data were collated using an Excel spreadsheet and analysed descriptively. For Likert responses, the data were reported in three categories: agree (strongly agree and agree responses), neutral (neither agree nor disagree responses) and disagree (disagree and strongly disagree responses). Frequencies, proportions and appropriate graphics were used to present the data.

### Ethical considerations

Ethical clearance to conduct this study was obtained from the University of Cape Town Faculty of Health Sciences Human Research Ethics Committee (reference no.: HREC 459/2022). This study complied with the Helsinki Declaration. Participants have been protected through strict processes of anonymisation of data, protection of their personal information, maintenance of confidentiality throughout the process and a no-risk guarantee of withdrawal from participation at all times. Identifying elements were removed from the dataset by the project lead before being shared with the rest of the project team unless the participant specifically indicated that these could be shared. All participants completed an online informed consent form prior to commencing with data submission.

## Results

**FIGURE 1:**
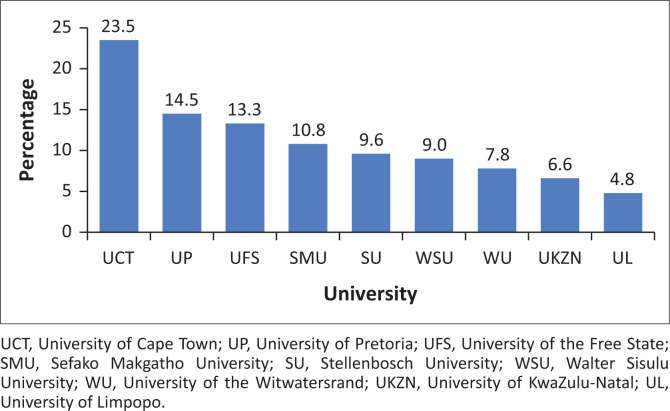
University affiliation of respondents.

A total of 166 invited participants completed the survey (76% of the intended sample size). They came from all nine health sciences faculties that provide specialist medical training in South Africa. The University of Cape Town (*n* = 39/166) yielded the most respondents and the University of Limpopo (*n* = 8/166) was least well represented (see Figure 1).

Fifty-one per cent (*n* = 85) of respondents were programme conveners and/or managers and/or directors and 48.8% were clinical and/or research supervisors. Respondents represented 53 of the 85 (62%) speciality and subspeciality designations admitted to the respective colleges of the CMSA. Most respondents (84.4%) had more than 2 years of experience; 48.2% had more than 10 years of experience; 17.5% had 6–10 years of experience; 18.7% had 2–5 years of experience and 15.7% had less than 2 years of experience.

Respondents were asked questions in four domains: knowledge of WBA and EPAs (nine items), existing practices aligned with WBA (eight items), existing practices related to decision-making (five items) and questions about how adequate the mechanisms of governance and support were for implementing WBA (three items) (see Online Appendix 1 for the survey questions).

### Knowledge of workplace-based assessment and entrustable professional activities

**FIGURE 2:**
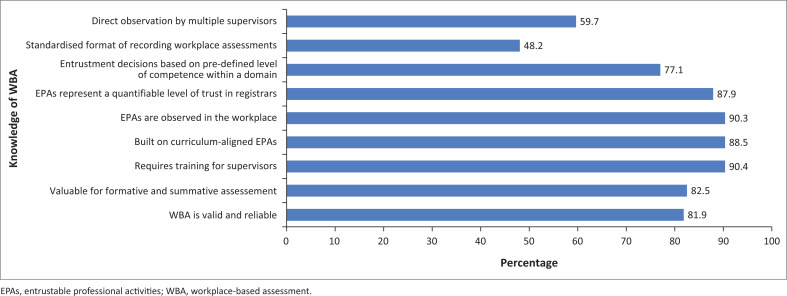
Knowledge of workplace-based assessment (including entrustable professional activities).

Respondents were asked nine questions to determine their self-assessed knowledge of WBA and EPAs. They were asked whether they perceive WBA as valid and reliable, whether WBA holds value for both formative and summative assessments, and whether it requires specialised training for supervisors. Furthermore, they were asked if WBA is based on a framework of curriculum-aligned EPAs, whether EPAs can be observed in the workplace, whether EPAs represent a quantifiable level of trust that the supervisor has in the trainee and whether EPAs should be ‘awarded’ only after a trainee has reached a pre-defined level of competence within a particular domain. Finally, they were also asked whether the recording of WBA should be standardised and whether WBA includes an emphasis on direct observation by *various* supervisors. Responses across answers were averaged under the rubric of knowledge of WBA (including EPAs) (Figure 2).

On average, 78.5% of respondents agreed with the nine knowledge-related statements regarding WBA and EPAs included in the survey. Only 10.6% were neutral about the statements, while 11% disagreed.

Respondents were asked how often they thought WBAs should take place, with possible answers of daily, weekly, monthly and quarterly. The majority (53%) indicated that assessments should occur more frequently than quarterly, while the remaining 47% felt that quarterly assessments were adequate.

### Current workplace-based assessment-aligned practices

**FIGURE 3:**
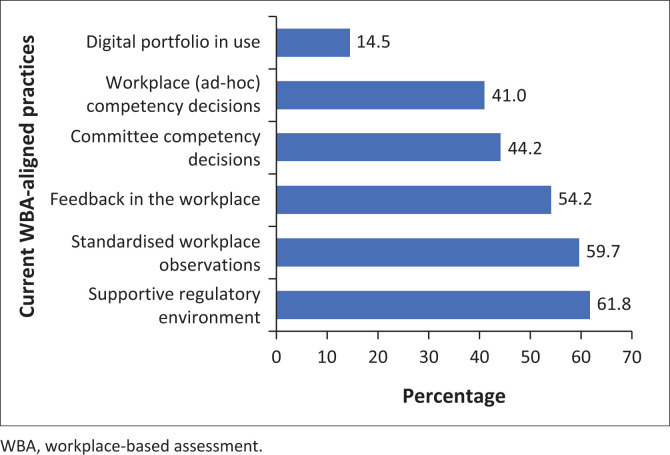
Current workplace-based assessment-aligned practices.

In surveying practices already implemented/aligned with WBA, respondents were asked about standardised workplace observations, provision of feedback and making standardised decisions about trainees’ level of competence. Figure 3 summarises these data.

The frequency of performing workplace observations using standardised tools was reported as follows: weekly (10.2%), monthly (12%), quarterly (38%), annually (11.4%) and not conducted at all (28.3%). A total of 48.2% of respondents reported using standardised workplace assessment tools, while 38.5% did not and 13.3% remained neutral. More than half of the respondents (59.7%) reported making direct observations of trainees in the workplace; specified standardised observation tools were not mentioned in this context. Furthermore, a similar proportion (54.2%) reported using feedback as part of a learning conversation in the workplace. The survey did not address issues related to the duration or quality of feedback.

Making ‘entrustment’ decisions about trainee competence is a core outcome of WBA. Less than half of the respondents (41%) reported current practices which included formal decisions about competence levels in the workplace, while the balance either remained neutral (22.9%) or indicated that this was not their current practice (35.5%). Just over half of the respondents (51.2%) indicated that faculty met regularly to discuss trainee progress, and 44% reported that these discussions were used to make decisions about trainee ‘competence and/or progression’.

### Staff readiness

Another key area influencing the delivery of WBA is staff capacity. Although this study did not measure the actual capacity for WBA delivery, respondents were asked to indicate their perceptions of staff readiness. Only 36.1% of respondents agreed that supervisors in their programmes were capable of meeting WBA expectations; 38% remained neutral, while 25.9% disagreed. Additionally, most respondents (90.4%) reported that supervisors needed specific training to be able to implement WBA effectively.

### Institutional support and resources

The institutional regulatory environment in which WBA is delivered is important because it provides a mandate for these activities. Respondents were asked whether there was adequate support and resources for implementing WBA, and 61.8% agreed that this was the case. In contrast, a minority (19.9%) disagreed, stating that adequate support and resources were not available.

### Workplace-based assessment records

The final domain covered by the survey addressed the issue of how WBA data were being managed within training programmes. Most respondents (41.6%) reported using paper-based WBA records; 28.3% reported a combination of paper and digital recordings and 14.5% kept digital WBA records only.

## Discussion

This cross-sectional, observational descriptive study provides insight into the current self-reported knowledge and practice of WBA among postgraduate specialist training programme directors and supervisors in South Africa. A key finding is the variable implementation despite relatively high levels of self-reported knowledge. This situational analysis should inform the national implementation plan being jointly spearheaded by local universities and CMSA governance structures. Figure 3 provides a summary that can be used as a baseline to track implementation progress over time.

The relatively high levels of self-reported knowledge of WBA concepts observed in this study align with findings in the literature. Anderson and colleagues reported varying levels of knowledge and perception, which are influenced by local contextual factors.^17^ The conceptual awareness of WBA could also be attributed to the possibility that respondents intuitively relate to these concepts. This could be attributed to workplace observations and assessments of competence already being an informal part of workplace supervision, that is clinical supervisors are already completing these tasks, although they may not be calling them ‘workplace-based assessments’. This is an important realisation because there is a risk that ‘educational supervision’ and workplace assessments may be seen as separate phenomena, resulting in a false perception of an impending additional burden on trainees and supervisors.^18^ In a resource-constrained environment such as South Africa, it is imperative that supervisors and trainees have a common understanding of how WBA will be formally integrated into the workplace.

This study has identified several areas for intervention in relation to rolling out a national formal programme of WBA in postgraduate specialist training in South Africa. This includes the need for developing standardised ways of observing trainees in the workplace, which assumes a uniform approach to benchmarking. When one considers the vast differences in training contexts, this concept becomes increasingly important. In anticipation of this, supervisors’ concerns have already been reported in the local context.^6^ Workplace-based assessment represents an opportunity to engage with inequities across a national training platform, providing data to leaders and planners that would allow for targeted interventions. In so doing, medical education can contribute to a social justice agenda within broader society.^19^

Similarly, providing constructive feedback to trainees within the context of a trusting supervisor-trainee relationship has been highlighted as a current deficiency. The importance of this relationship as a platform for learning cannot be underestimated.^20^ When feedback using culturally appropriate techniques is employed within this relationship, learning is enhanced.^21^

Supervisor training needs to be culturally and contextually sensitive, particularly in the resource-rich and culturally diverse training environments typical of South Africa. Other important interventions include finding ways to enhance assessment literacy among supervisors, which could pave the way to improved workplace competency decision-making, constituting CCCs, developing and implementing cost-effective digital learning portfolios, and reviewing university regulations to ensure that WBA is included in institutional policy frameworks. These have all been shown to be core elements of a sustainable WBA programme.^22^

It is worth comparing the early South African experience reported here with findings from elsewhere in the Global South. Singapore began integrating WBA into their local medical education curricula in the late 2000s.^23^ There was a rapid and dramatic shift in 2010 from a traditional process-based model with high-stakes summative assessments to a competency-based postgraduate training model^24^ that ultimately integrated WBA into both formative and summative assessment processes. Like South Africa, the health system dynamics in Singapore differed significantly from the United States (US).^24^ The shift was viewed, as it is by some clinicians in South Africa, as unrealistic from the perspective of having inadequate resources to support and sustain it. However, over a decade later, Singapore has succeeded.^25^ The alignment between SACOMD, the CMSA and health professions education researchers provides the groundswell for similar optimism, though a medium-long-term view may be needed when measuring success.

### Limitations

This study has several limitations. Firstly, the use of survey methodology meant that uninterested parties, who may have legitimate concerns about the implementation of WBA, were not included, which introduces sampling bias because of non-response. These potentially dissenting voices are important as they may anticipate risks that may not be recognised by supporters of WBA implementation. Secondly, trainees and other key stakeholders in WBA, such as health facility managers, were not included in the study. Their perspectives are important because the power differential between supervisors and trainees may result in deferential behaviour among trainees. This is a disadvantage as trainee perspectives are essential for co-creating a strategy for sustainable systemic innovation. Thirdly, the use of self-reported data regarding knowledge and practice is challenging for many reasons already articulated in the literature.^26^ Lastly, while the process of face and content validation was completed, this study did not engage in construct validation or reliability testing and so cannot comment on the internal consistency of the data collection tool.

## Conclusion

We surveyed postgraduate specialist training programme directors and supervisors in an LMIC context regarding their self-reported knowledge and practice of WBA. Knowledge levels were high, but current practice in the workplace was varied. These findings highlight key areas to focus on when developing and implementing a comprehensive WBA rollout strategy in South Africa. Future research should focus on the supervisor–trainee relationship, culturally sensitive methods of feedback, the use of critical reflection as a learning method in training and the system requirements needed to sustain WBA in an LMIC.
